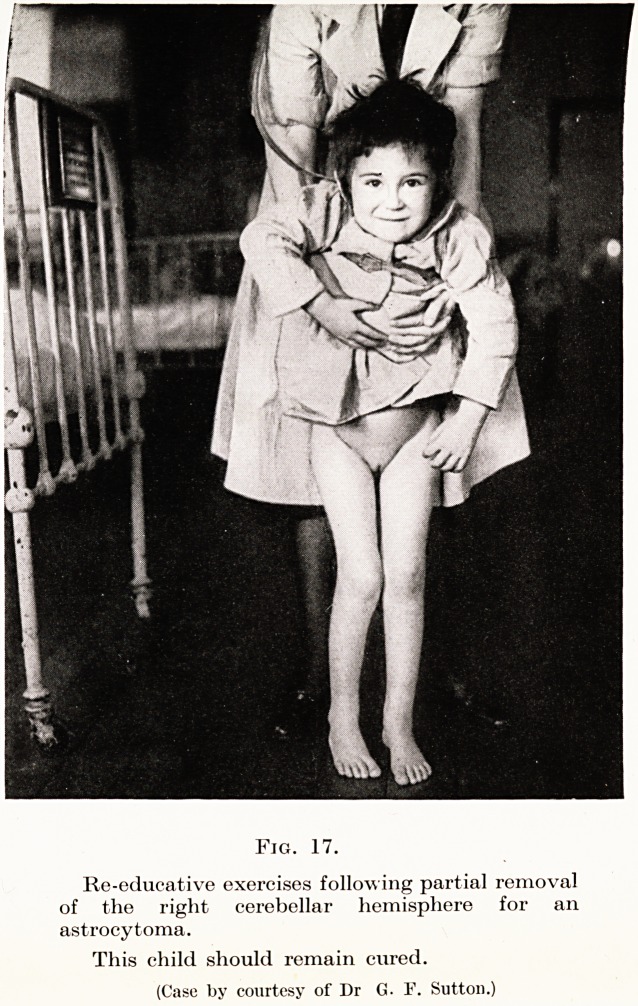# Some Problems in the Diagnosis and Treatment of Intra-Cranial Tumours

**Published:** 1938

**Authors:** F. Wilfred Willway

**Affiliations:** Assistant Surgeon West End Hospital for Nervous Diseases; Surgical Registrar Bristol Royal Infirmary


### The Bristol Medico-Chirurgical Journal

*" Scire est nescire, nisi id me
Scire alius sciret."*

AUTUMN, 1938.

### SOME PROBLEMS IN THE DIAGNOSIS AND TREATMENT OF INTRA-CRANIAL TUMOURS.

BY

F. Wilfred Willway, M.D., M.S., B.Sc., F.R.C.S.

*Assistant Surgeon West End Hospital for Nervous Diseases ;
Surgical Registrar Bristol Royal Infirmary.*

Three main questions have to be answered in any suspected case of intra-cranial tumour. In some cases the answers are obvious, while in others even the most elaborate means of investigation may fail to provide a full answer. Yet the neuro-surgeon's aim should be to provide full answers where possible, as only then is he in a position to decide the best line of treatment.

1. Is there a tumour ?
2. Where is the tumour ?
3. What is the nature of the tumour ?

In reference to this article a " tumour " is considered as any " space-occupying lesion " and, of course, may vary from the most benign cyst, through

L

the low-grade malignancy tumours, to neoplasms of a very high-degree malignancy. The term "space-occupying lesion" is, however, somewhat clumsy, and the word "tumour" will be used instead in this paper.

The classical signs of an intra-cranial tumour as detailed in many medical text-books are :—headache, vomiting and papillœdema. It cannot be over-emphasized that these are not the symptoms of a tumour *per se*, but those of a raised intra-cranial pressure (often from a tumour) and are usually signs of an advanced state of affairs. It is true that in some cases raised tension occurs early and in others that one or other of the classical signs appear from other causes. The important point is that the practitioner should not wait for these signs before referring his patient to the specialist. He might as well refuse to diagnose appendicitis until there is generalized abdominal rigidity, or cancer of the breast until there is loss of weight.

This point is stressed because raised tension increases the surgeon's difficulties in diagnosis and treatment, and vastly increases the risks of operation.

A parallel might well be drawn with senile enlargement of the prostate, thus :—

| | |
|---|---|
| Senile Enlargement of the Prostate. | Intra-Cranial Tumour. |
| \| | \| |
| Local Urinary Dysfunction. | Local Neural Dysfunction. |
| \| | \| |
| General Urinary Dysfunction. Uræmia. | General Intra-Cranial Pressure. |
| \| | \| |
| Coma and Death. | Coma and Death. |

The true signs of a tumour are the signs of local deterioration of cerebral function, a tumour being essentially a destructive agent. "Cerebral insults" is a useful descriptive term for many of these attacks of destruction of function. To these insults may be added epileptiform attacks. These two signs form the essential features of an intra-cranial tumour. As, however, many of these tumours lie in silent areas of the brain, the essential focal symptoms may not be apparent until signs of increased tension are well marked.

The diagnosis of an intra-cranial lesion follows the general principles of medical investigation, thus :—

Suspicion.

Investigation.

| Refutation. | Ambiguity. | Confirmation. |
| --- | --- | --- |
| Discharge. | Observation. | Treatment. |
| | Repeated Examination. | |

Clearly the problem for the practitioner is one of suspicion. All vague, odd neurological cases must be suspect. Migraine, fits, strokes, giddiness, failing vision, hysteria, etc., should all be considered as potential tumour cases and at least worthy of investigation.

### METHODS OF INVESTIGATION.

These may be summarized in the following manner :—

1. General neurological examination.
2. Examination of the fields of vision. Perimetry.
3. Manometric and laboratory examination of the cerebro-spinal fluid.
4. Plain and stereoscopic X-ray views.
5. Encephalography.
6. Ventriculography.
7. Arteriography.
8. Electroencephalography.

Roughly speaking, it may be said that the first three are the province of the neurologist and of the pathologist, the last five the province of the radiologist and of the neuro-surgeon. It is not proposed to deal further with the first three except to acknowledge their paramount importance, and to stress that the other investigations are rightly termed accessory investigations. One point may, however, be stressed—the use of the manometer in lumbar puncture. This little instrument is an essential adjunct to lumbar puncture. To estimate cerebro-spinal pressure by allowing fluid to drop from a lumbar puncture needle is about as accurate and scientific as estimating the red cell count by looking at the lower conjunctival recess; and alas, it is far more dangerous. Manometry is simple, safe and informative, and should be the routine procedure. (Fig. 1.)

Plain X-rays do not commonly give direct information as to the situation of cerebral tumours. Much indirect evidence is, however, often afforded. Hammer markings of the skull, separation of the sutures, alterations of the clinoid processes and sella turcica, erosion of bone, hyperostosis, alterations in vascular

FIG. 1.
The correct way to perform lumbar puncture, *i.e.*, with a manometer.

FIG. 2.
Intra-cranial calcification in a supra-sellar cyst. Successful relief of symptoms by operation.

(Case by courtesy of Dr. Orr Ewing. Skiagram by courtesy of Dr. Bush.)

### PLATE VII

Fig. 3.

Unilateral calcification in the choroid plexus, embedded in a huge glioblastoma multiforme.

(Specimen by courtesy of Dr. Fraser.)

Fig. 4.

Ventriculogram.

Intra-ventricular tumour, fourth ventricle. Note the distended aqueduct of sylvius and the air around the shadow in the fourth ventricle.

(Film by Dr. Mayes. Author's case, with Dr. Todd.)

markings, changes in the optic foramina and internal auditory meatus, may all hold peculiar significance. (Fig. 2.) Of especial interest is pineal calcification. Over the age of forty years most pineal glands are sufficiently calcified to be radiopaque. The shadow is normally strictly midline. Deviations to right or left surely indicate a lesion on the opposite side. There is, however, a pitfall here in that spontaneous calcification occasionally occurs in the choroid plexus in the vestibular region. This is normally bilateral and about an inch from the midline. When unilateral (as in a recent case) confusion may occur, and what is assumed to be a calcified pineal displaced from the midline is really choroid plexus. (Fig. 3). In the majority of cases, however, location will not yet have been obtained and more exact accessory measures are required.

### ENCEPHALOGRAPHY AND VENTRICULOGRAPHY.

With very few exceptions, "space-occupying lesions" within the cranial cavity distort or displace the ventricular system. Normally the ventricular cavities and their contents are of the same degree of radio-translucency as the brain, and thus do not appear on an X-ray. If, however, the cerebro-spinal fluid be withdrawn and replaced by air a contrast is obtained, and the ventricular outlines may be studied. The fluid may be drawn off and air replaced in either of two ways : via the lumbar subarachnoid puncture—encephalography ; or by direct ventricular tap—ventriculography. Encephalography can only be employed when there is no rise of intra-cranial tension ; if any real rise be present encephalography is far too dangerous. The method has the advantage of not being an "operation" and is simple, but it is unfortunately an extremely unpleasant experience for the patient. Severe headache, nausea, vomiting and some collapse almost always occur and may last for two or three days.

Ventriculography is more of an operation, in that a burr hole has to be made in the skull. (Fig. 4.) Except in extreme cases, however, the after effects are not so unpleasant. If there is much tension and much ventricular displacement, there may be a sudden exacerbation of symptoms after ventriculography, and this operation must never be performed unless the surgeon is prepared to proceed to a decompression if necessary. (Figs. 5 to 8.) There is an increasing tendency to make the ventriculography the first stage in the treatment of any case, and as soon as the films have been studied to proceed to the major operation without delay. It is hoped in this way to avoid unpleasant reactions.

### A MAJOR NEURO-SURGICAL PROBLEM.

"To refuse the evil and choose the good."—*Isa.* vii. 16.

Selection of cases based on pre-operative information is the ideal of the neuro-surgeon, but frequently he has to fall far short of this aim and to perform exploratory operations. Here the difficulties are manifest. No surgeon performs a laparotomy for carcinoma of the stomach when he can feel metastases in the liver, or amputation of the breast when there are secondary deposits in the spine. No surgeon performs a resection of gut and anastomosis in the

### PLATE VIII

FIG. 5.

FIG. 6.

FIG. 7.

FIG. 8.

Ventriculograms showing symmetrical hydrocephalus in a child with a cerebellar tumour. The child after operation. Note the filling of the third ventricle.

(Author's case. Films by Dr. Orley.)

PLATE IX

FIG. 9.

FIG. 10.

The right and the wrong way to deal with irremoveable tumours.

1. *Wrong.* Huge decompression cerebral hernia rapidly developing. (Patient was aphasic and remained so after the operation for six months.)

2. *Right.* The bone flap is replaced, the scar in the hair line, and no one could tell he has had a brain operation.

presence of intestinal obstruction (if strangulation be excepted). He will drain the bowel and at a later date perform his resection. No surgeon removes the prostate in the presence of uræmia: he will drain the bladder and await improvement. But in analogy this is precisely what the neuro-surgeon may have to do. He cannot with sufficient accuracy determine whether his case is inoperable until he has operated, and thus the position is too late for a retracement of steps. Most neuro-surgeons are agreed that tumours of the type glioblastoma multiforme are so malignant, so liable to recur locally after free removal, that knowingly they will not operate on these cases. The maximum survival period is three to four months after such operations, and their performance is probably not justifiable. If an operation is performed and the tumour is not removed an enormous cerebral hernia develops, and the patient leads an unhappy, partially paralysed, vegetable type of existence. In fact he may suffer more from his cerebral hernia than from the original rapidly developing tumour. (Fig. 9.)

If the skull has been opened and such a tumour found the best course is free excision until the bone flap can be replaced firmly without tension, and the patient thus secured against a hernia. (Fig. 10.) If the tumour is in an active area of the brain such extirpation will lead to pitiful paralysis. Thus it can be readily seen that the surgeon's best course is to refuse these bad cases if he can detect them. There is the rub. In the absence of exact preoperative knowledge one is bound to explore on the chance that the tumour is more benign in nature. If the surgeon does not explore he may watch his "hopeless" case die and then find that the patient had a relatively benign and accessible lesion.

### CAN FURTHER EXACT KNOWLEDGE BE OBTAINED ?

Two other preoperative investigations are possible : arteriography and electroencephalography. The latter examination lies outside the detailed knowledge of the writer, but it is certain that it can throw no light on the nature of the underlying lesion. Electroencephalography gives with considerable exactness information as to damage to cortical function, but no information as to the cause of the damage, which may be vascular disease, inflammatory lesion or tumour.

Arteriography, however, is worthy of further consideration. Intra-cranial tumours displace and distort the cerebral blood vessels, and information as to localization may be obtained from a study of such displacement. (Figs. 11 to 14). But, further, the tumour may have a circulation peculiar to itself of new-formed vascular spaces, or the tumour may alter the normal circulation in some intimate way so that the actual pathology of the tumour may be predicted. Of course, this is not regularly possible, but it is sufficiently frequent to make the method worthy of use and further study. Meningiomas, angiomas and aneurysms may be diagnosed by this means with confidence. This is a valuable step, as the meningiomas are an exceedingly favourable class, while an aneurysm does not require an intra-cranial operation, but ligature of the corresponding carotid artery.

Briefly, arteriography consists in X-ray visualization

PLATE X

FIG. 11.

Normal arteriogram. Retouched to show the principal vessels.

(Author's case. By courtesy of Dr. Clarke. Film kindly retouched by Mr. W. A. Jackman.)

FIG. 12.

Arteriograms.

1. *Above.* Hydrocephalus from subtentorial tumour.

2. *Below.* Frontal tumour. Note the distortion of the vessels.

Fig. 13.

Arteriogram (*below*) and phlebogram (*above*) from a case of meningioma. Note how the tumour is outlined.

(By courtesy of Dr S. Lima.)

Fig. 14.

Aneurysm, indicated by arrow, of the anterior communicating artery.

(Author's case.)

Arteriogram from a similar case.

(By courtesy of Dr. S. Lima.)

of the cerebral vessels after the injection of radi-opaque thorotrast into the carotid. Its advantage compared with ventriculography is that it does not affect intra-cranial tension and so is less upsetting to the patient. Its major disadvantage is that thorotrast is not inert but is radio-active, and is not eliminated by the body but concentrated in the reticulo-endothelial system. Experimental work shows that it is to some degree carcinogenic; but it must be remembered that in such work the doses are far in excess of those used in this investigation. In selected cases the advantages of the method outweigh its limitations.

Most of the space of this article has been devoted to problems of diagnosis rather than of treatment. This is because many of the problems of treatment are too technical to be of general interest: the real problems are those of exact diagnosis.

### PROGNOSIS.

Mortality rate figures may indicate the skill of a surgeon, or merely his timidity or indeed temerity. To open the skull and not to open the dura mater, for example, can hardly be termed an intra-cranial operation: such a surgeon's mortality rate would be negligible, as would be the benefit obtained in a large series of cases. Intra-cranial tumours cannot be considered as a whole. Sir David Ferrier in 1898 wrote: "The treatment of intra-cranial tumours forms a rather melancholy chapter in therapeutics." With regard to the gliomata, especially those of the glioblastoma multiforme type, this statement is still true. It is by no means true of pituitary tumours, suprapituitary tumours, meningiomas, auditory neurinomas, cerebellar hæmangiomatous cysts, chronic arachnoiditis and the like. (Figs. 15 to 17.)

The bold neuro-surgeon will lose many of his patients, but he will have more real cures. Of the surviving bad cases one may say that the end results should be about as good as for operations for cancer of the stomach, though the operations are more desperate and accompanied by greater risk.

PLATE XII

FIG. 15.

FIG. 16.

Case of meningioma of right frontal region. Successful removal. Photograph on eighth post-operative day.

Tumour from similar case. Fresh weight 3½ oz.

(Author's case. By courtesy of Dr. H. Carleton.)

FIG. 17.

Re-educative exercises following partial removal of the right cerebellar hemisphere for an astrocytoma.

This child should remain cured.

(Case by courtesy of Dr G. F. Sutton.)

## Figures and Tables

**Fig. 1. f1:**
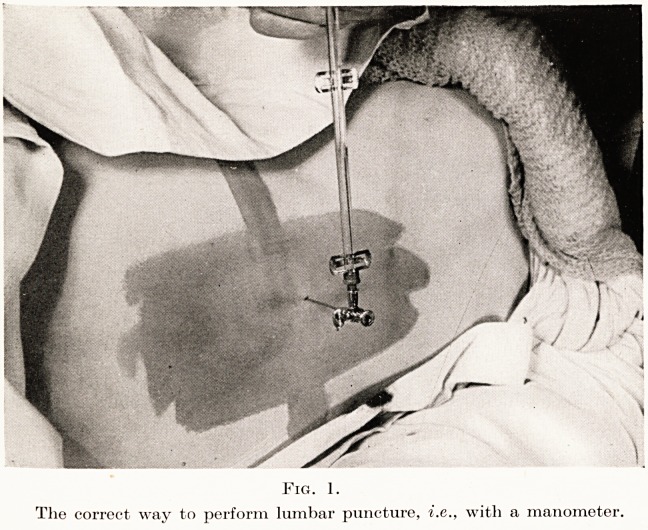


**Fig. 2. f2:**
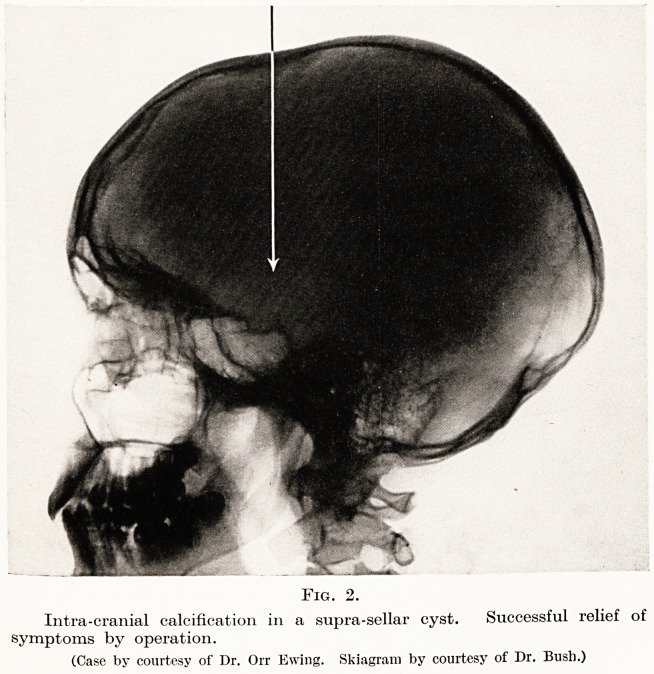


**Fig. 3. f3:**
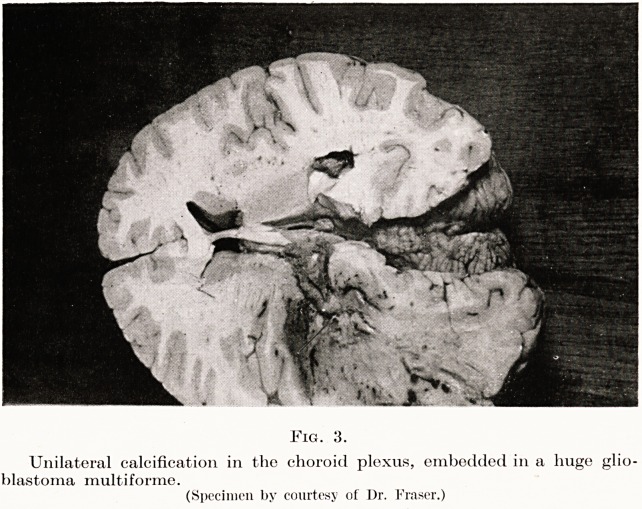


**Fig. 4. f4:**
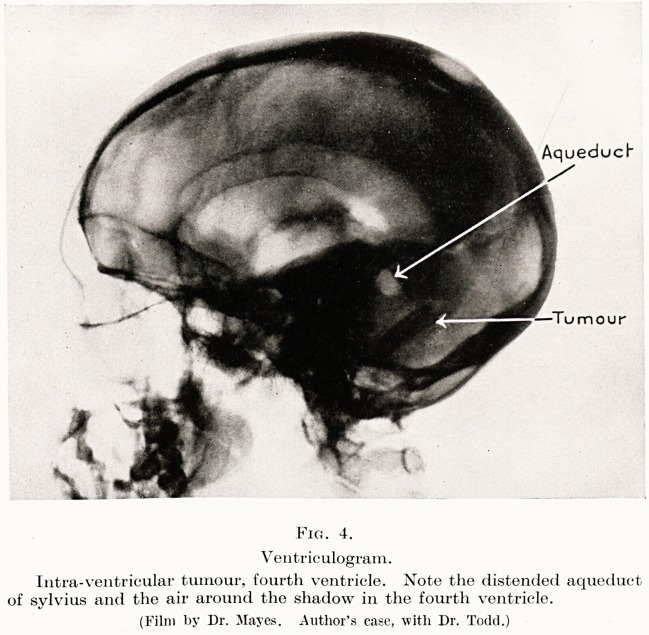


**Fig. 5. Fig. 6. Fig. 7. Fig. 8. f5:**
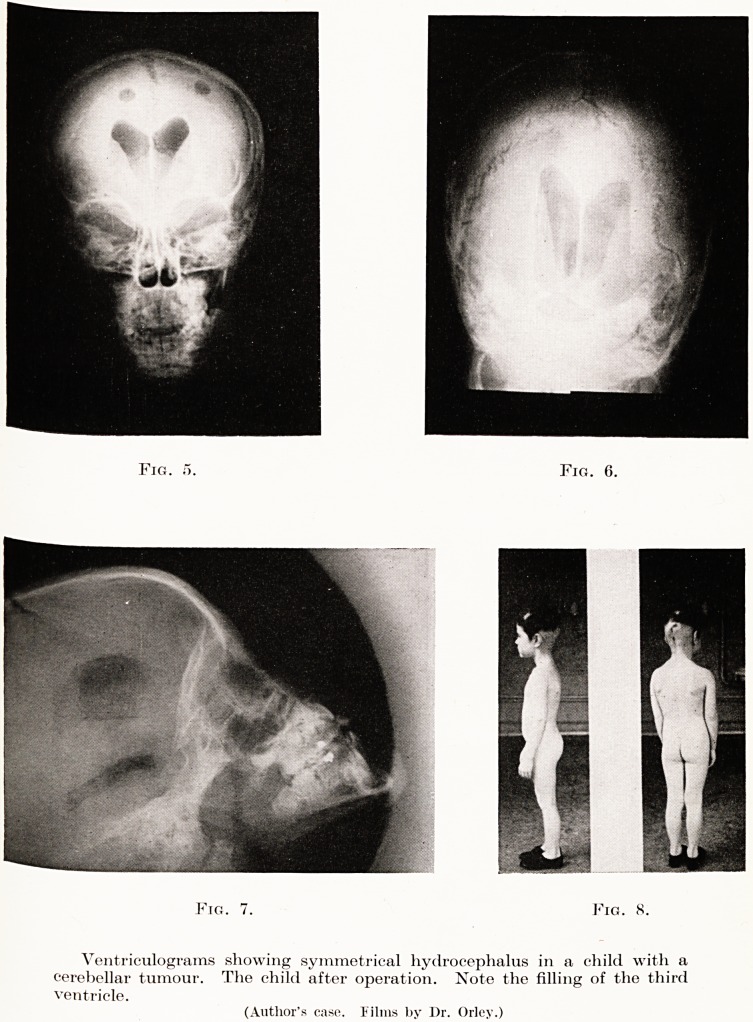


**Fig. 9. Fig. 10. f6:**
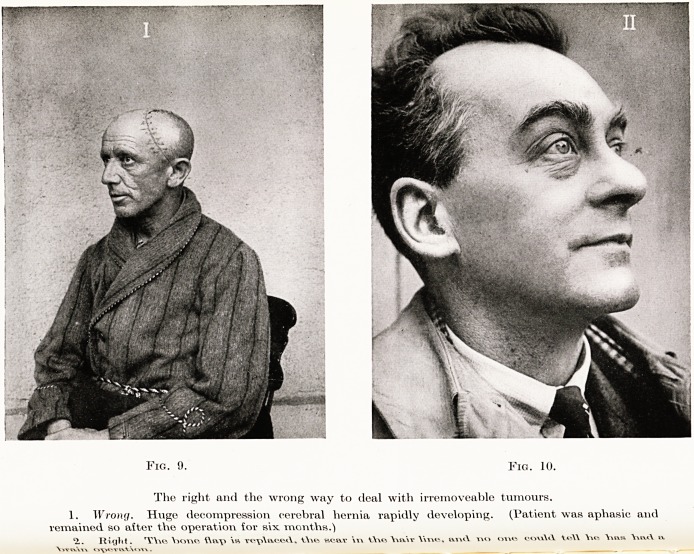


**Fig. 11. f7:**
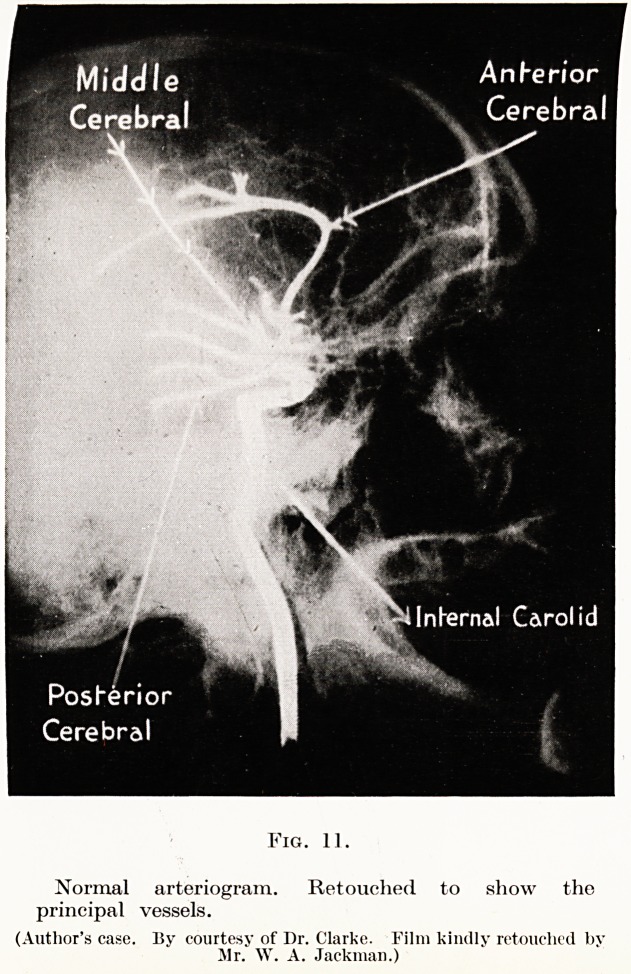


**Fig. 12. f8:**
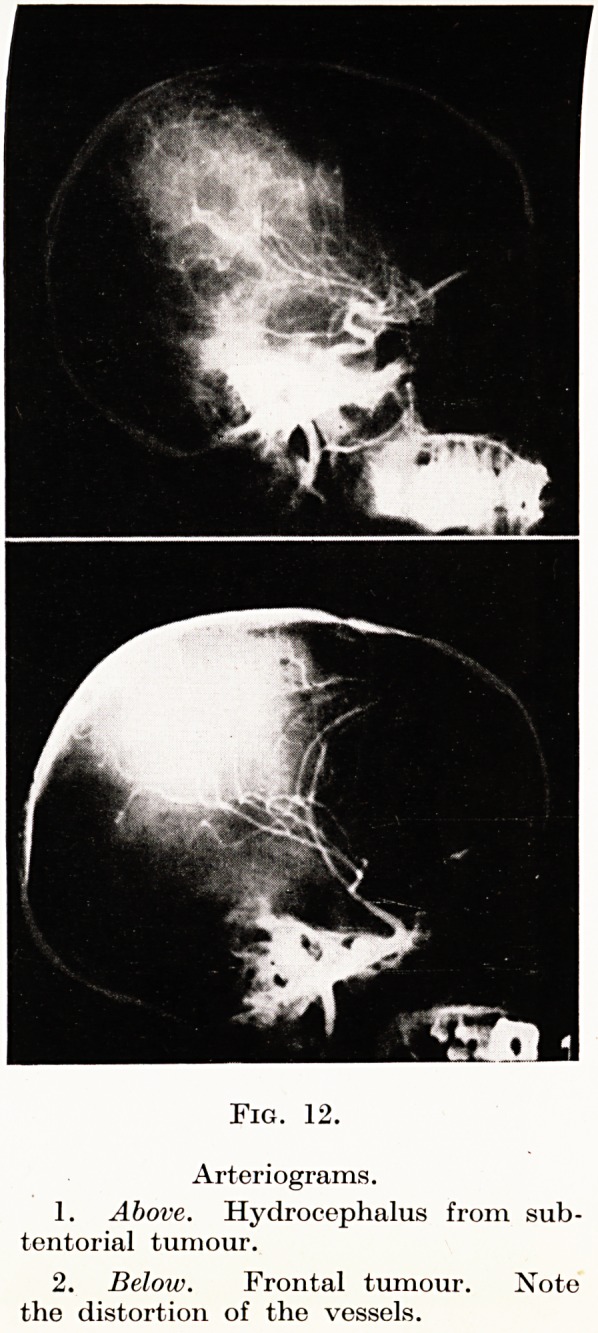


**Fig. 13. f9:**
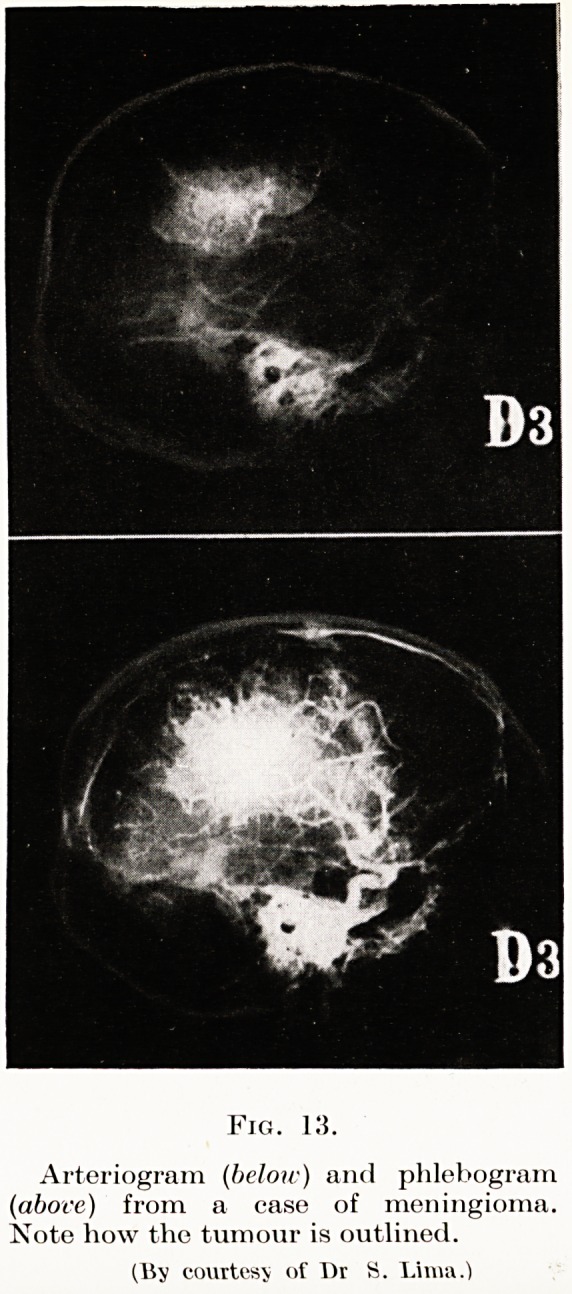


**Fig. 14. f10:**
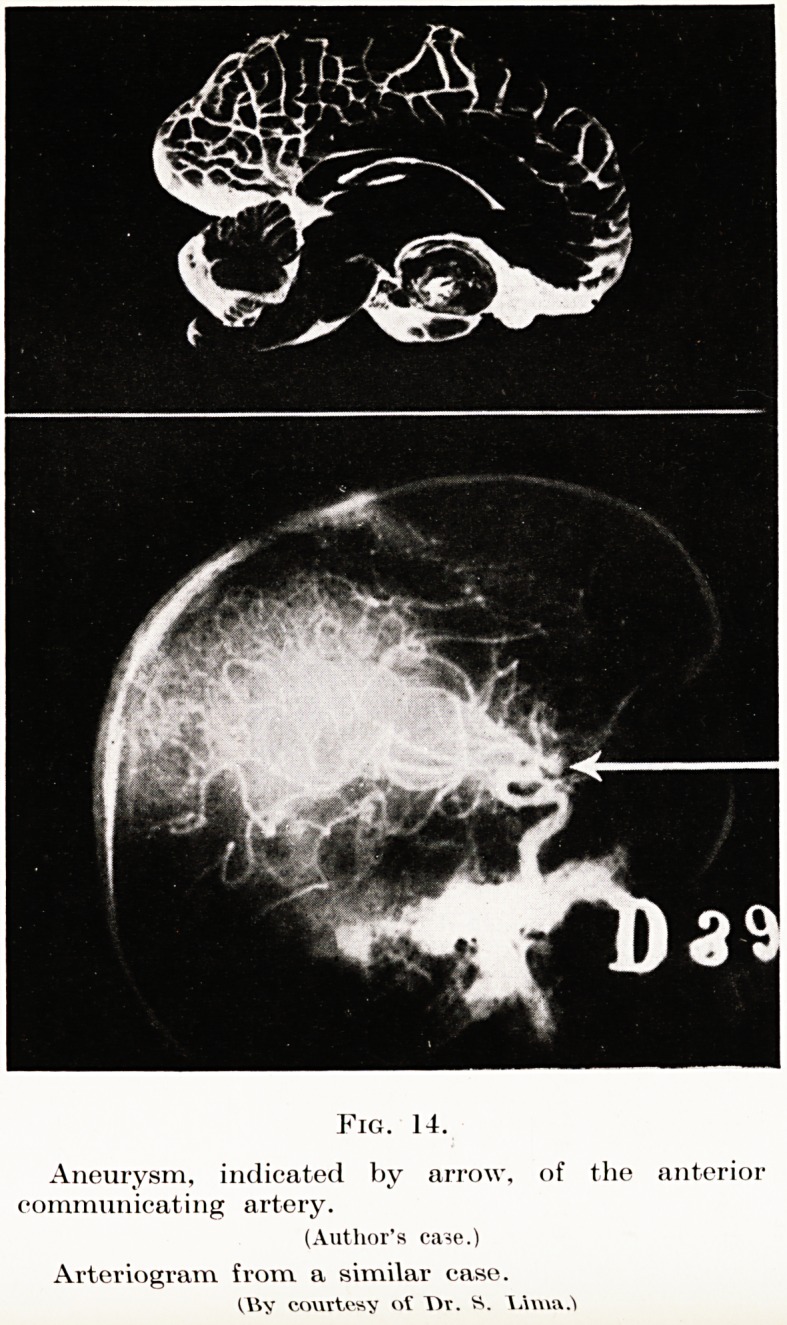


**Fig. 15. f11:**
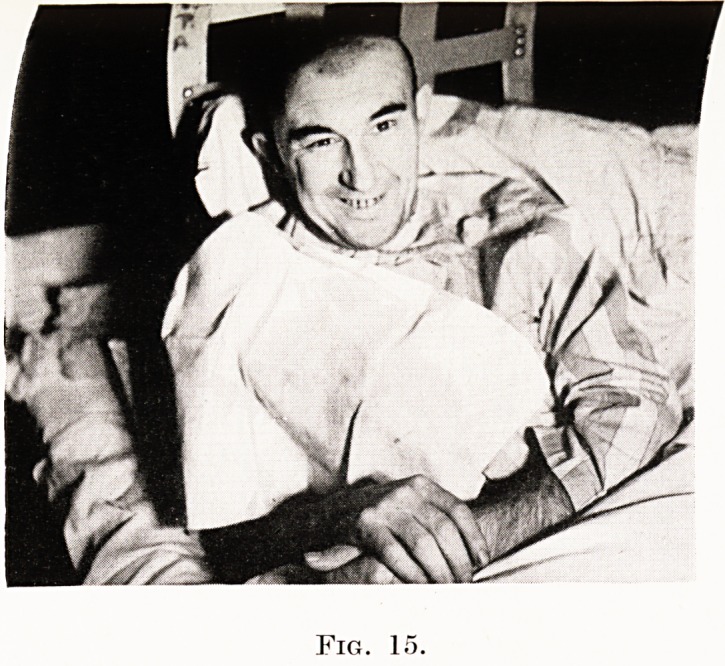


**Fig. 16. f12:**
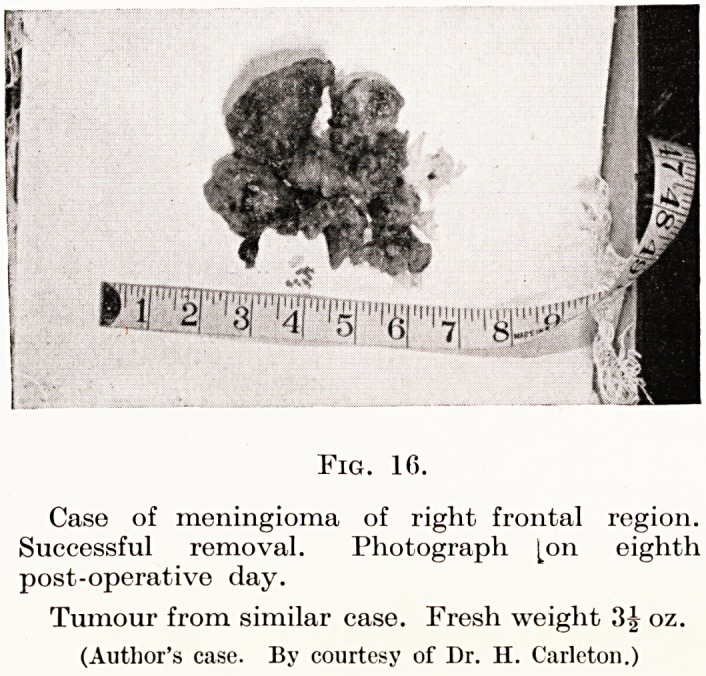


**Fig. 17. f13:**